# An Analytical Survey of Trace Heavy Elements in Insecticides

**DOI:** 10.1155/2019/8150793

**Published:** 2019-05-29

**Authors:** Maha Abdallah Alnuwaiser

**Affiliations:** Department of Chemistry, College of Science, Princess Nourah bint Abdulrahman University, P.O. Box 28445, Riyadh 11437, Saudi Arabia

## Abstract

There are many types of insecticides traded in the local and international markets, which vary depending on the type of target insect (e.g., whether crawling or flying). This paper aimed to assess the concentration of trace elements present in the various pesticide formulations (solid, liquid, and gaseous). This study was conducted in two groups: the first group was comprised of zinc, copper, iron, chromium, phosphorus, selenium, and cobalt; the second group included four heavy toxic elements (arsenic, thallium, lead, and mercury). These elements were analyzed by inductively coupled plasma/optical emission spectrometry (ICP-OES).

## 1. Introduction

With the growth of the world population, food production will need to increase by more than 60%, assisted by the safe and effective use of pesticides. Pesticides have an important role in helping to eliminate insects that destroy crops or cause epidemics diseases [1–5].

The increase in the use of pesticides often results in many adverse impacts, especially on the agricultural environment [6, 7]. For this reason, various analytical techniques are used to test pesticides, including chromatographic methods [8, 9]. Pesticides are widely used [10] in agriculture, medicine, and industry and have the potential to change the ecosystem [11]. Pesticides contain active ingredients and are used to ensure that high agricultural yield and quality can be achieved, but can cause environmental pollution if used incorrectly [12]. Organophosphorus pesticides (e.g., tebufenozide, chromafenozide, methoxyfenozide, and acetonitrile) [13] target those pests affecting fruit and vegetable crops, such as apples, grapes, cucumbers, cabbage, tomatoes, and spinach [14].

Some pesticides have large effects on mental and reproductive health and the developmental neural degenerative diseases among pregnant women and children [1, 4, 9]. If the percentage of pesticide toxicity dose increases, it leads to asthma and anaphylaxis in the human nervous system [9, 15].

Pesticide alternatives often reduces the need for pesticides, including systemic insecticides, which are highly effective in the elimination of crop pests, using biological soil, pest-resistant tools, and fatty acid salts (potassium salts) [16]. Pyrethroids are used as an alternative to highly toxic pesticides and are inexpensive [17, 18]. Neonicotinoids, alternatives to pesticides, are widely used in agriculture and are highly water-soluble [19]. Many pesticides and heavy metals are durable and nonbiodegradable and can accumulate along biological chains (soil, plants, food, and seawater) [20]. Therefore, the presence of large amounts of pesticides and heavy metals in the environment represents a risk to human health and the environment. For this reason, accurate monitoring of these concentrations plays an important role [21]. The literature cites many methods for heavy metal determination in soils, phosphorus rocks, seawater, plants, biologic materials, steel, and cast iron, including inductive coupled plasma-mass spectrometry [22], inductive coupled plasma atomic emission spectrometry [23], atomic absorption spectrometry with flame or electrothermal atomization [24], electrochemically with ultramicroelectrodes [25], and anodic stripping voltammetry [26].

For these reasons, we chose to determine the concentrations of heavy metals and pesticides in insecticides. This work investigates trace (zinc, copper, iron, chromium, phosphorus, selenium, and cobalt) and heavy metal (arsenic, thallium, lead, and mercury) pollution of pesticides. The trace and heavy metals were analyzed using inductively coupled plasma/optical emission spectrometry (ICP-OES).

## 2. Materials and Methods

### 2.1. The Samples and Their Sources

In October 2018, sixteen commonly available pesticides samples were collected from the local markets in Riyadh, Saudi Arabia. These samples cover the two types of exterminating insect (crawls and flying) and were classified into three major groups depending on the physical statement (solid, liquid, and gaseous). The first group consists of six solid pesticide samples [Acefed powder (mithomail), Probalt wettable powder (aimida klobrid), Nur cam 5 de-powder cypermethrin), over 50000 de me a powder (Malathion), and Cockroach powder (Deltamethrin)]. The second group included six liquid pesticide samples [Cyper Safe aqueous, a microscopic effective cypermethrin; Cyber art, a 100 Torrent AC emulsification liquid with active substance cypermethrin; Sniper-Center, a commentator fluid with active substance fipronil; Scope 60 w, an odorless liquid with active substance Asaybrmthrin; Brodeur 20%, a DSL liquid soluble in water with active ingredient aimida klobrid; and Rajesh 25, an AC liquid EC with active ingredient Deltamethrin]. The third group has four gas pesticide samples (Pif Paf for all bugs, with active substances imiprothrin and transfluthrin; Pigeon flies and mosquitoes with the active substances phenothrin and permethrin; Pigeon for cockroaches and ants with active substances pyrethroid and cypermethrin and imiprothrin; and Raid for all insects, with active substance imiprothrin and cyfluthrin).

### 2.2. Preparation of Samples

In the case of solid samples, 1.0 g was dissolved in 10 mL distilled water and filtered. For the liquid samples, 1.0 g of each sample was mixed with distilled water to a final volume of 10 mL. For the gaseous samples, the aerosols were collected from the packets by spraying in the separation funnel, and equal amounts of distilled water were added. The mixtures were shaken well and allowed to separate overnight. The aqueous layer was then isolated from the funnel and filtered.

#### 2.2.1. Precautions

No heating or acid digestion was performed due to the volatility of the samples. To accurately determine the dissolved elements, the samples were filtered using a 0.45-*μ*m membrane.

### 2.3. Instruments

Measurements of the trace and heavy elements were performed using a Perkin Elmer ICP-OES Optima 7300 DV Spectrometer.

*Operating Conditions: *plasma gas flow, 15 L/min; auxiliary gas flow, 0.2 L/min; nebulizer gas flow, 0.6 L/min; RF power, 1450 watts; plasma view axial read parameters, 2.0 min, 5.0 max; peristaltic pump flow rate, 1.5 mL/min; number of replicates, 3; resolution, normal; aqueous torch assembly.

Wavelengths used for the elements: arsenic(As), 193.696; chromium(Cr), 205.560; cobalt(Co), 228.616; copper(Cu), 324.752; iron(Fe), 239.562; lead(Pb), 220.353; mercury(Hg), 253.652; phosphorous(P), 213.617; selenium(Se), 203.985; thallium(Tl), 276.787; Zinc (Zn), 213.857.

### 2.4. Data Analysis and Calculations

The measurement units for the assessed samples (solid, liquid, and aerosol) are microgram per liter (ug/L) or Parts Per Billion (ppb). For trace elements with zero ppb, these were considered as “nondetectable”, meaning that the analyte concentration/intensity was negative or that the analyte concentration is below the method's detection limit.

## 3. Results and Discussion

**Figure 1:**
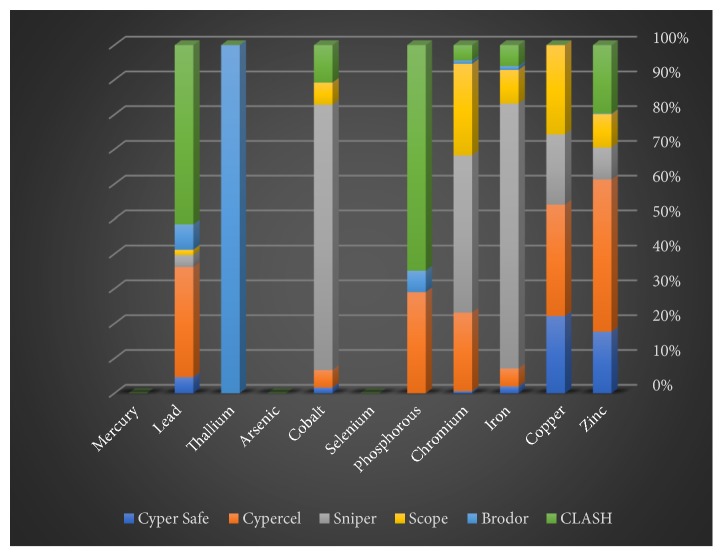
Proportions of the studied elements in the liquid insecticides samples.

**Figure 2:**
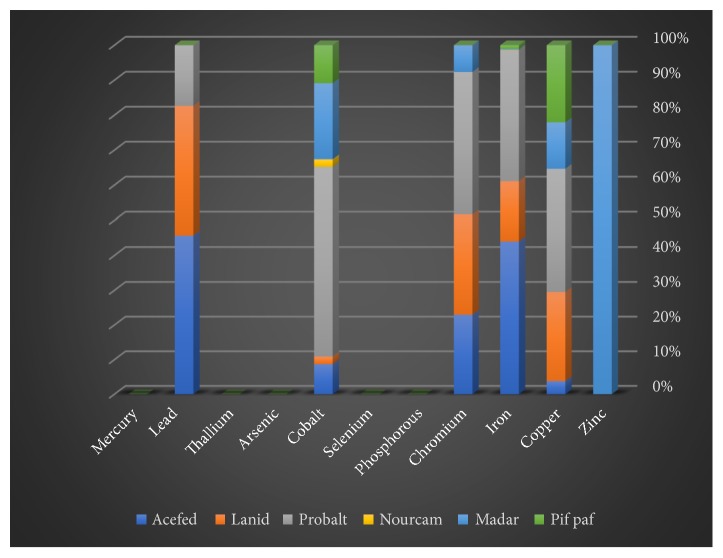
Proportions of the studied elements in solid insecticides samples.

**Figure 3:**
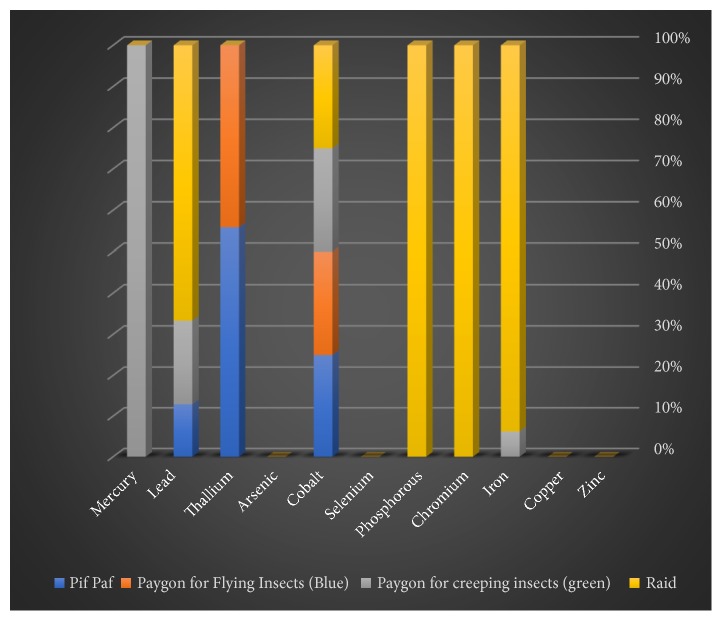
Proportions of the studied elements in the gaseous insecticides samples.

Some of the trace (zinc, copper, iron, chromium, phosphorus, selenium, and cobalt) and heavy (arsenic, thallium, lead, and mercury) elements in solid, liquid, and gaseous pesticides species were determined by spectrometry. This study focused on the three insecticide categories consisting of six liquid, six solid, and four gaseous samples. Among the liquid insecticides samples, selenium, arsenic, and mercury were not detected (Table 1 and Figure 1). The concentrations of the other elements (Zn, Cu, Fe, Cr, P, Co, Tl, and Pb) varied. Among the solid pesticide samples, zinc, phosphorus, selenium, arsenic, thallium, and mercury were absent (Table 2 and Figure 2). On the other hand, it was found that most of the samples of gaseous pesticides are free of all studied elements, and some of them contain only two to four elements (Table 3 and Figure 3).

The zinc, copper, iron, chromium, phosphorus, cobalt, thallium, and lead elements were detected in all liquid insecticide samples (Table 1). Among the liquid insecticides, the zinc concentration was highest in CyperCel (2389 ppb) and Clash (1078ppb) and lowest in Brodor (10ppb). Copper was detected in four of the liquid pesticide samples [Cyper Safe (464 ppb), CyperCel (669 ppb), Sniper (423 ppb), and Scope (539 ppb)]. The iron content was high in the Sniper pesticide (46,190 ppb) in case of the six types of liquid insecticides. The percentage of chromium element was increased in case of the three insecticide samples (Sniper (746 ppb), Scope (437 ppb), and CyperCel (373 ppb)) but it has the lowest concentration in case of the two pesticide samples (Cyper Safe (10 ppb) and Brodor (16 ppb)), respectively. Among the liquid insecticide samples, the phosphorus concentration was high in Clash (842 ppb), but was not detected in Cyper Safe, Sniper, and Scope. The cobalt concentrations were as follows: Sniper (275 ppb), Clash (39 ppb), Scope (23 ppb), and CyperCel (18 ppb). Thallium was present only in the liquid Brodor sample (92ppb). Among the liquid insecticides, lead was detected in (from the highest to the lowest) Clash (1316 ppb), CyperCel (807 ppb), Brodor (186 ppb), Cyper Safe (119 ppb), Sniper (88 ppb), and Scope (39 ppb).

Zinc, copper, iron, chromium, cobalt, and lead elements were detected in all of the solid insecticide samples (Table 2). Among the solid insecticide samples, the lowest detectable concentration of zinc was found in Madar (10 ppb). Zinc was absent from the solid samples of Acefed, Lanid, Probalt, Nourcam, and Pif Paf. The percentages of copper element in case of the three solid pesticide Lanid, Probalt, and Pif Paf samples are 128 ppb, 179 ppb, and 110 ppb, respectively. The highest concentration value of copper element is represented in case of the Probalt pesticide and decreases in case of the Acefed (19 ppb) and Madar (66 ppb) but has a nil percentage in case of Nourcam sample. The highest percentage of iron element was observed in case of the Acefed pesticide (4298 ppb) compared with all the elements in this study which was applied on the six types of solid insecticides. The percentages of the chromium element for the three insecticide samples of Probalt, Lanid, and Acefed are 85 ppb, 60 ppb, and 60 ppb, respectively, but in case of Madar pesticide sample, the concentration is 16 ppb. Cobalt was detected in Acefed (4 ppb), Lanid (1 ppb), Probalt (25 ppb), Nourcam (1 ppb), Madar (10ppb), and Pif Paf (5 ppb), respectively. In the solid insecticides, lead was detected in (from highest to the lowest) Acefed (121 ppb), Lanid (98 ppb), and Probalt (46 ppb).

Zn, Fe, Cr, P, Co, Tl, and Pb were detected in the four gaseous pesticide samples (Table 3). Zinc element was found only in one insecticide sample (Paygon for creeping insects, green, 52 ppb). The iron content was highest in the Raid insecticide (150 ppb) and lowest in Paygon for creeping insects (green). Both chromium and phosphorus were detected only in the Raid insecticide (33 and 20 ppb, respectively). Thallium was detected in two samples [Pif Paf (19 ppb) and Paygon for flying insects (blue)]. The lead concentration was highest in the case of Raid (62 ppb), but was not detected for Paygon for flying insects (blue).

The impacts of the active substance in different insecticides and in different kinds of insecticide (liquid-solid, solid-gas, solid-liquid) are listed in Table 4.

*(i) Active Substances in the Similar Insecticide Types. *Among the liquid insecticide samples, the zinc concentration was highest in CyperCel (2389 ppb) and Cyper Safe (968 ppb), lowest in Paygon for creeping insects (green) (52 ppb), and absent from the solid insecticides. The percentages of copper element are 464 ppb and 669 ppb in case of the two liquid pesticide samples Cyper Safe and CyperCel, respectively. The concentration of copper became lower in case of the solid samples Acefed (19 ppb) and Lanid (128 ppb), respectively, but the copper ratio is nil for the gaseous samples. The concentration of iron element is increased in both liquid and solid insecticide samples, while it has a nil or 10 ppb concentration in the Pif Paf and Paygon gaseous pesticide samples, respectively. The percentage of chromium element is detected in case of the liquid and solid pesticide samples with different ratios, but it has a nil ratio in gaseous samples. The concentration of phosphorus ratio is nil in case of solid and gaseous pesticide samples, while it was presented in one of the liquid insecticide samples, CyperCel (377 ppb). Selenium and mercury elements were detected in none of the three pesticide types (liquid, solid, and gas). Cobalt was present in the three samples at 1-18 ppb. Thallium was detected in one of the gaseous samples (Pif Paf, 19 ppb). Lead was present in the three cases of pesticide samples with different ratios. It has a higher concentration in case of the liquid samples than in solid insecticide samples, while it was present in gaseous samples with lower ratio.

*(ii) Active Substances in the Different Insecticide Types. *The highest percentage of zinc element is presented in case of Clash (1078 ppb) liquid insecticide in comparison with Pif Paf solid insecticide sample. The highest ratio of copper element is present in case of Probalt (179 ppb) solid insecticide sample in comparison with a liquid sample (Brodor, nil percentage). The iron ratio was higher in the solid sample (3655 ppb, Probalt) than in the liquid sample (664 ppb, Brodor) and, paradoxically, the ratio of iron in liquid Clash was higher than in Pif Paf solid (3676 ppb vs. 102 ppb). The chromium ratio has a 85 ppb in case of Probalt solid insecticide sample. This ratio is higher than liquid Brodor insecticide sample (16 ppb). Paradoxically, it was found that the ratio of chromium in case of liquid Clash sample (73 ppb) is higher than solid Pif Paf sample (nil). The phosphorus element ratio is only present in case of Brodor and Clash liquid insecticide samples as 80 ppb and 842 ppb, respectively. The cobalt element is present in case of the Clash liquid sample (39 ppb) with moderate ratio but it has a lower ratio in case of the solid and gaseous samples. The percentages of selenium, arsenic, and mercury elements are nil in case of the three (liquid-solid, solid-gas, solid-liquid) samples. The thallium ratio is only found in one liquid sample (Brodor, moderate ratio, 92 ppb). Lead element was present in the three cases with different ratios. It has a higher concentration in case of the Clash liquid sample (1316 ppb) rather than the solid and gaseous insecticide samples.

The studied insecticides were divided into three groups: insect insecticides, insecticides, and insecticides for all types of insects. For the tested creeping insect insecticides, Sniper had the highest concentration of elements (Table 5). For flying insect insecticides, the liquid Brodor insecticide contained a higher concentration of elements than the biodegradable insecticide. Also, we found that the liquid insecticides contained higher concentrations of elements than the solid and gaseous pesticides. When comparing insecticides from the same case and product, it can be concluded that the Paygon for insects is more likely to occur in the appearance and concentration of elements. When comparing the different physical formulations, the Pif Paf solid concentrates contained copper and iron elements rather than the Pif Paf gas vaporizer samples. We tested the effect of the odor ratio on pesticides using two types of liquid and cruciferous insecticides (of the same composition and active substance). Compared to CyperCel odor, the element concentrations were lower in the Cybersif syrup insecticide.

## Figures and Tables

**Table 1:** The percentage (ppb) of elements under study in liquid insecticides samples.

Elements		Liquid pesticides samples					
		Cyper Safe	CyperCel	Sniper	Scope	Brodor	CLASH
Basic elements	Zinc	968	2389	506	527	10	1078
	Copper	464	669	423	539	0	0
	Iron	1202	3117	46190	5892	664	3676
	Chromium	10	373	746	437	16	73
	Phosphorous	0	377	0	0	80	842
	Selenium	0	0	0	0	0	0
	Cobalt	6	18	275	23	0	39

Toxic elements	Arsenic	0	0	0	0	0	0
	Thallium	0	0	0	0	92	0
	Lead	119	807	88	39	186	1316
	Mercury	0	0	0	0	0	0

**Table 2:** The percentage (ppb) elements in the solid insecticides samples.

Elements		Solid pesticides samples					
		Acefed	Lanid	Probalt	Nourcam	Madar	Pif Paf
Basic elements	Zinc	0	0	0	0	10	0
	Copper	19	128	179	0	66	110
	Iron	4298	1675	3655	13	29	102
	Chromium	48	60	85	0	16	0
	Phosphorous	0	0	0	0	0	0
	Selenium	0	0	0	0	0	0
	Cobalt	4	1	25	1	10	5

Toxic elements	Arsenic	0	0	0	0	0	0
	Thallium	0	0	0	0	0	0
	Lead	121	98	46	0	0	0
	Mercury	0	0	0	0	0	0

**Table 3:** The percentage (ppb) elements in the gaseous insecticides samples.

Elements		Gaseous pesticides samples			
		Pif Paf	Paygon for Flying Insects (Blue)	Paygon for creeping insects (green)	Raid
Basic elements	Zinc	0	0	52	0
	Copper	0	0	0	0
	Iron	0	0	10	150
	Chromium	0	0	0	33
	Phosphorous	0	0	0	20
	Selenium	0	0	0	0
	Cobalt	1	1	1	1

Toxic elements	Arsenic	0	0	0	0
	Thallium	19	15	0	0
	Lead	12	0	19	62
	Mercury	0	0	0	0

**Table 4:** The impact of the active substance in different insecticides.

Elements	Active substance common in similar types of insecticides						Active substance common in different kinds of insecticides					
	Cyber mthrin		Methomyl		Imiprothrin		Aimida klobrid		Cyber mthrin		Deltamethrin	
	Cyper Safe	CyperCel	Acefed	Lanid	Pif Paf Gas	Paygon for creeping insects (green)	Brodor	Probalt	Nurcam	Paygon for creeping insects (green)	Pif Paf solid	clash
Zinc	968	2389	0	0	0	52	10	0	0	52	0	1078
Copper	464	669	19	128	0	0	0	179	0	0	110	0
Iron	1202	3117	4298	1675	0	10	664	3655	13	10	102	3676
Chromium	10	373	48	60	0	0	16	85	0	0	0	73
Phosphorous	0	377	0	0	0	0	80	0	0	0	0	842
Selenium	0	0	0	0	0	0	0	0	0	0	0	0
Cobalt	6	18	4	1	1	1	0	25	1	1	5	39

Arsenic	0	0	0	0	0	0	0	0	0	0	0	0
Thallium	0	0	0	0	19	0	92	0	0	0	0	0
Lead	119	807	121	98	12	19	186	46	0	19	0	1316
Mercury	0	0	0	0	0	0	0	0	0	0	0	0

**Table 5:** Comparison between the pesticides of all bugs for flying and crawling insects.

Elements		Paygon for creeping insects (green)	Sniper	Pif Paf solid	Madar	Nourcam			Brodor	Paygon for Flying Insects (Blue)
Zinc	Creeping insects	52	506	0	10	0	Flying insects		10	0
Copper		0	423	110	66	0			0	0
Iron		10	46190	102	29	13			664	0
Chromium		0	746	0	16	0			16	0
Phosphorous		0	0	0	0	0			80	0
Selenium		0	0	0	0	0			0	0
Cobalt		1	275	5	10	1			0	1

Arsenic	Creeping insects	0	0	0	0	0	Flying insects		0	0
Thallium		0	0	0	0	0			92	15
Lead		19	88	0	0	0			186	0
Mercury		0	0	0	0	0			0	0

The elements		Lanid	Acefed	Probalt	Raid	Pif Paf Gas	clash	CyperCel	Cyper Safe	Scope

Zinc	All types of insects	0	0	0	0	0	1078	2389	968	527
Copper		128	19	179	0	0	0	669	464	539
Iron		1675	4298	3655	150	0	3676	3117	1202	5892
Chromium		60	48	85	33	0	73	373	10	437
Phosphorous		0	0	0	20	0	842	377	0	0
Selenium		0	0	0	0	0	0	0	0	0
Cobalt		1	4	25	1	1	39	18	6	23

Arsenic	All types of insects	0	0	0	0	0	0	0	0	0
Thallium		0	0	0	0	19	0	0	0	0
Lead		98	121	46	62	12	1316	807	119	39
Mercury		0	0	0	0	0	0	0	0	0

## Data Availability

The data used to support the findings of this study are available from the author upon request.
